# Optimizing Laparoscopic Cholecystectomy in Aberrant Biliary Anatomy: A Case of Cystic Duct Insertion Into the Right Posterior Hepatic Duct

**DOI:** 10.7759/cureus.81483

**Published:** 2025-03-30

**Authors:** Kim-Long Le, Minh-Quang Tran, Phu-Cuong Pham, My-Tran Trinh, Tri-Nhan Pham

**Affiliations:** 1 Hepato-Pancreato-Biliary Surgery Department, Nhan dan Gia Dinh Hospital, Ho Chi Minh, VNM; 2 Surgery Department, Pham Ngoc Thach University of Medicine, Ho Chi Minh, VNM

**Keywords:** biliary anatomical variation, critical view of safety, cystic duct variant, intraoperative cholangiography, laparoscopic cholecystectomy, right posterior hepatic duct

## Abstract

Anatomic variations of the biliary tree, particularly those involving the cystic duct, pose significant challenges during laparoscopic cholecystectomy and can lead to inadvertent bile duct injury (BDI) if unrecognized. We report a rare variant in which the cystic duct inserts into the right posterior hepatic duct, which itself drains directly into the common bile duct - an aberrant configuration not frequently described in the literature. A 65-year-old man with grade I acute cholecystitis underwent preoperative imaging with computed tomography and magnetic resonance cholangiopancreatography, revealing the unusual biliary anatomy. A laparoscopic cholecystectomy was performed using the critical view of safety (CVS) approach, followed by intraoperative cholangiography (IOC) via gallbladder puncture to delineate the anatomy, ensuring accurate identification of the cystic duct and artery. The procedure was completed safely without complications. This case highlights the importance of preoperative imaging, adherence to CVS, and IOC in achieving safe outcomes in the setting of complex biliary anatomy. Despite increasing interest in indocyanine green (ICG) fluorescence cholangiography, its availability remains limited in some centers; thus, conventional techniques remain valuable. We advocate for a multimodal approach to mitigate the risk of BDI in patients with atypical biliary configurations.

## Introduction

Laparoscopic cholecystectomy has been widely recognized as the gold standard for managing gallbladder diseases due to its minimally invasive nature and favorable recovery profile. However, the risk of bile duct injury (BDI), a severe complication with significant morbidity, persists, particularly in cases involving complex anatomical variations of the biliary tree. Among these, anomalies such as aberrant connections between the cystic duct and right posterior hepatic duct (RPHD) are exceedingly rare, complicating intraoperative navigation and increasing the likelihood of misidentification during dissection [1-3].

Effective preoperative and intraoperative strategies are imperative for mitigating such risks. Magnetic resonance cholangiopancreatography (MRCP) has emerged as a vital diagnostic tool in delineating biliary anatomy preoperatively​ [4]. Intraoperatively, the critical view of safety (CVS) and intraoperative cholangiography (IOC) play central roles in preventing BDI. Briefly, CVS involves careful dissection of the hepatocystic triangle to clearly identify and separate the cystic duct and artery before any transection, whereas IOC entails injecting contrast through the cystic duct or gallbladder to visualize the biliary tree in real time. These methods are well established in reducing anatomical uncertainty, even in challenging variations. Furthermore, emerging modalities like indocyanine green (ICG) fluorescence cholangiography show promise in real-time visualization of biliary anatomy, though their utility remains underexplored in settings without widespread adoption [5].

In this report, we present a unique case involving the cystic duct draining into an aberrant RPHD. The clinical significance of this anatomical variation is highlighted alongside a discussion of the surgical strategies employed, including the application of CVS and IOC. This case underscores the importance of a systematic, multidisciplinary approach in managing rare biliary anomalies to ensure patient safety and surgical success.

## Case presentation

A 65-year-old male patient presented to the emergency department with a sudden onset of epigastric pain that had been persistent and non-relieving for several hours. The pain was described as severe and constant, radiating to the back, and was associated with mild nausea. The patient denied any history of vomiting, fever, or jaundice. He reported a similar, albeit milder, episode a few months prior that resolved spontaneously. There was no significant history of alcohol consumption or prior biliary symptoms.

Past medical history

The patient had a history of coronary artery disease, for which he underwent stenting in 2022. He was being treated for heart failure with reduced ejection fraction and was on regular medications, including Duoplavin, Bisoprolol, Valsartan, and Lipostatin. His family and personal history were otherwise unremarkable.

Physical examination

On admission, the patient was alert and oriented, with stable vital signs as follows: heart rate of 80 beats per minute, blood pressure of 120/70 mmHg, respiratory rate of 20 breaths per minute, and oxygen saturation of 99% on room air. His temperature was normal at 37.0°C. Abdominal examination revealed a soft abdomen with mild tenderness in the epigastrium but no guarding or rebound tenderness. No palpable masses were noted, and Murphy’s sign was negative.

Laboratory investigations

Initial laboratory investigations revealed leukocytosis with neutrophilia (WBC 11.54 × 10^9^/L, neutrophils 10.12 × 10^9^/L) and lymphopenia (lymphocytes 0.80 × 10^9^/L). Serum amylase was within normal limits (287 U/L; reference range: 73-393 U/L). Liver function tests showed mildly elevated direct bilirubin levels (5.82 µmol/L), with significantly elevated transaminases: aspartate aminotransferase (AST) 128 IU/L and alanine aminotransferase (ALT) 130 IU/L, underscoring the potential for a biliary or hepatocellular process. Coagulation profile was normal (prothrombin time (PT) 12.0 seconds, INR 1.09). Serum glucose, creatinine, and electrolytes were within reference ranges (Table 1).

**Table 1:** Relevant laboratory findings on admission Arrows indicate values outside the normal reference range: ↑ indicates an increase and ↓ indicates a decrease INR: international normalized ratio; AST: aspartate aminotransferase; ALT: alanine aminotransferase; HDL-cholesterol: high-density lipoprotein cholesterol; LDL-cholesterol: low-density lipoprotein cholesterol

Parameter	Result	Reference range
Hematology		
White blood cells (WBCs)	11.54 K/uL ↑	4.0-10.0 K/uL
Neutrophil	10.12 K/uL ↑	2.00-7.50 K/uL
Lymphocyte	0.80 K/uL ↓	1.00-3.50 K/uL
Hemoglobin (Hb)	131 g/L ↓	140-160 g/L
Hematocrit	0.440 L/L	0.35-0.47 L/L
Platelets (PLT)	167 Giga/L	150-400 Giga/L
Coagulation		
Prothrombin time (PT)	12.0 seconds	9.4-13.0 seconds
INR	1.09	0.8-1.2
Activated partial thromboplastin time (APTT)	29.3 seconds	25.4-36.9 seconds
Biochemistry		
Glucose	4.60 mmol/L	3.9-6.1 mmol/L
Creatinine	82.21 µmol/L	62-106 µmol/L
Sodium (Na)	139.8 mmol/L	135-145 mmol/L
Potassium (K)	4.06 mmol/L	3.5-5.0 mmol/L
Total bilirubin	16.39 µmol/L	≤17 µmol/L
Direct bilirubin	5.82 µmol/L ↑	≤5 µmol/L
AST	128 IU/L ↑	13-39 IU/L
ALT	130 IU/L ↑	7-52 IU/L
Cholesterol	3.12 mmol/L ↓	3.9-5.2 mmol/L
Triglycerides	0.57 mmol/L	0.46-1.88 mmol/L
HDL-cholesterol	1.15 mmol/L	≥0.9 mmol/L
LDL-cholesterol	1.67 mmol/L	≤3.4 mmol/L
Amylase	287 U/L	73-393 U/L

Imaging studies

**Figure 1:**
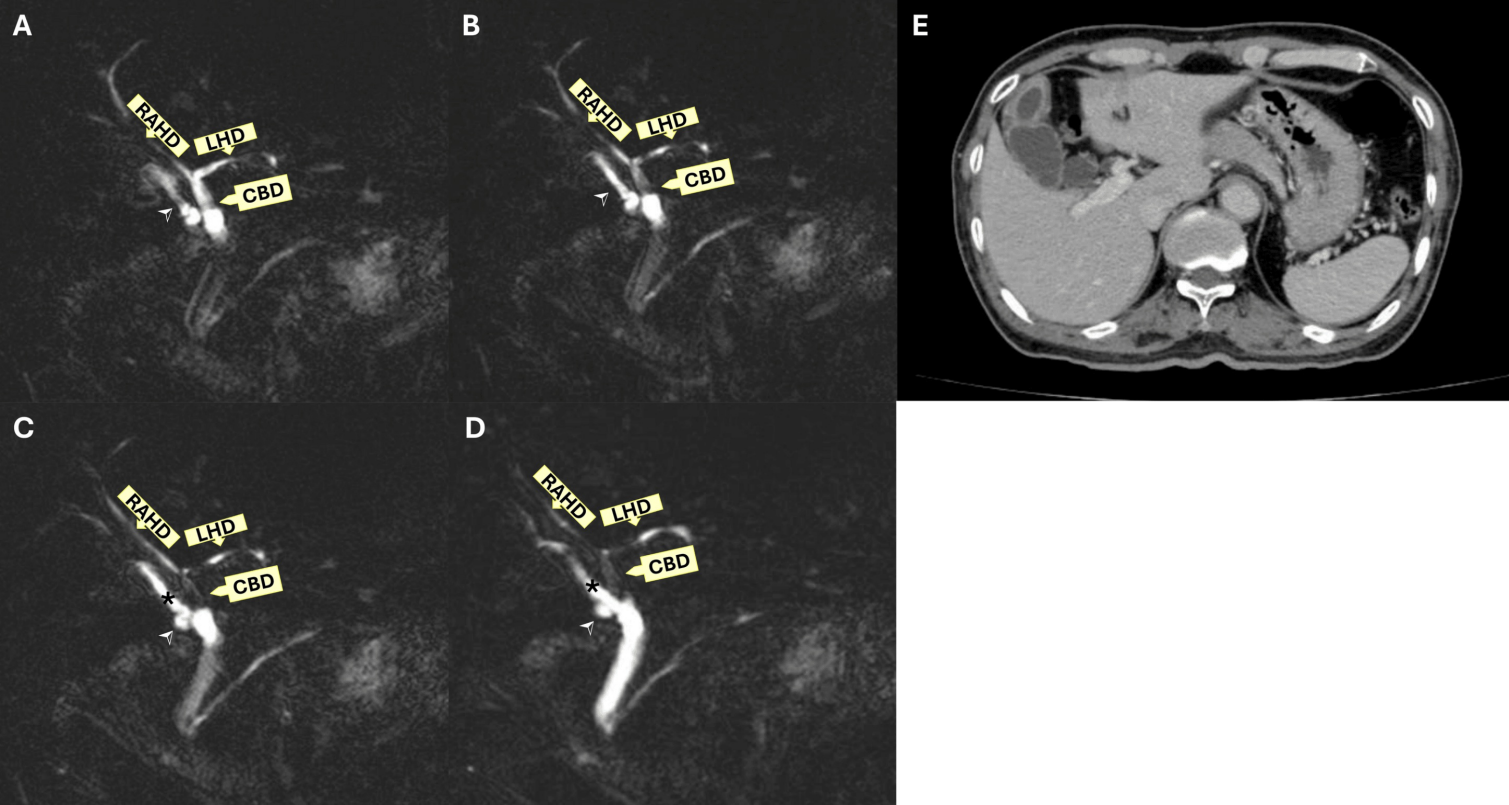
Preoperative images showing a variant of the right posterior hepatic duct A, B, C, D: Magnetic resonance cholangiopancreatography (MRCP) show the cystic duct (arrowhead) confluent to the variant of the RPHD (asterisk) that drains directly into the common bile duct (CBD). E: Axial CT scan demonstrates localized mural thickening of the gallbladder wall, predominantly at the fundus and body. There is no evidence of pericholecystic fat stranding. Both intrahepatic and extrahepatic bile ducts are not dilated. RPHD: right posterior hepatic duct; RAHD: right anterior hepatic duct; LHD: left hepatic duct

Abdominal ultrasound revealed a distended gallbladder with multiple gallstones and mild wall thickening but no pericholecystic fluid or signs of cholecystitis. The common bile duct was of normal caliber without visible stones. To confirm the diagnosis and rule out biliary obstruction or other complications, a computed tomography (CT) scan of the abdomen was performed. CT findings confirmed acute pancreatitis with no evidence of pancreatic necrosis or pseudocyst formation. MRCP revealed no choledocholithiasis but identified an anatomical variation: the cystic duct was draining into the RPHD, a rare biliary anomaly (Figure 1).

Management and surgical intervention

**Figure 2:**
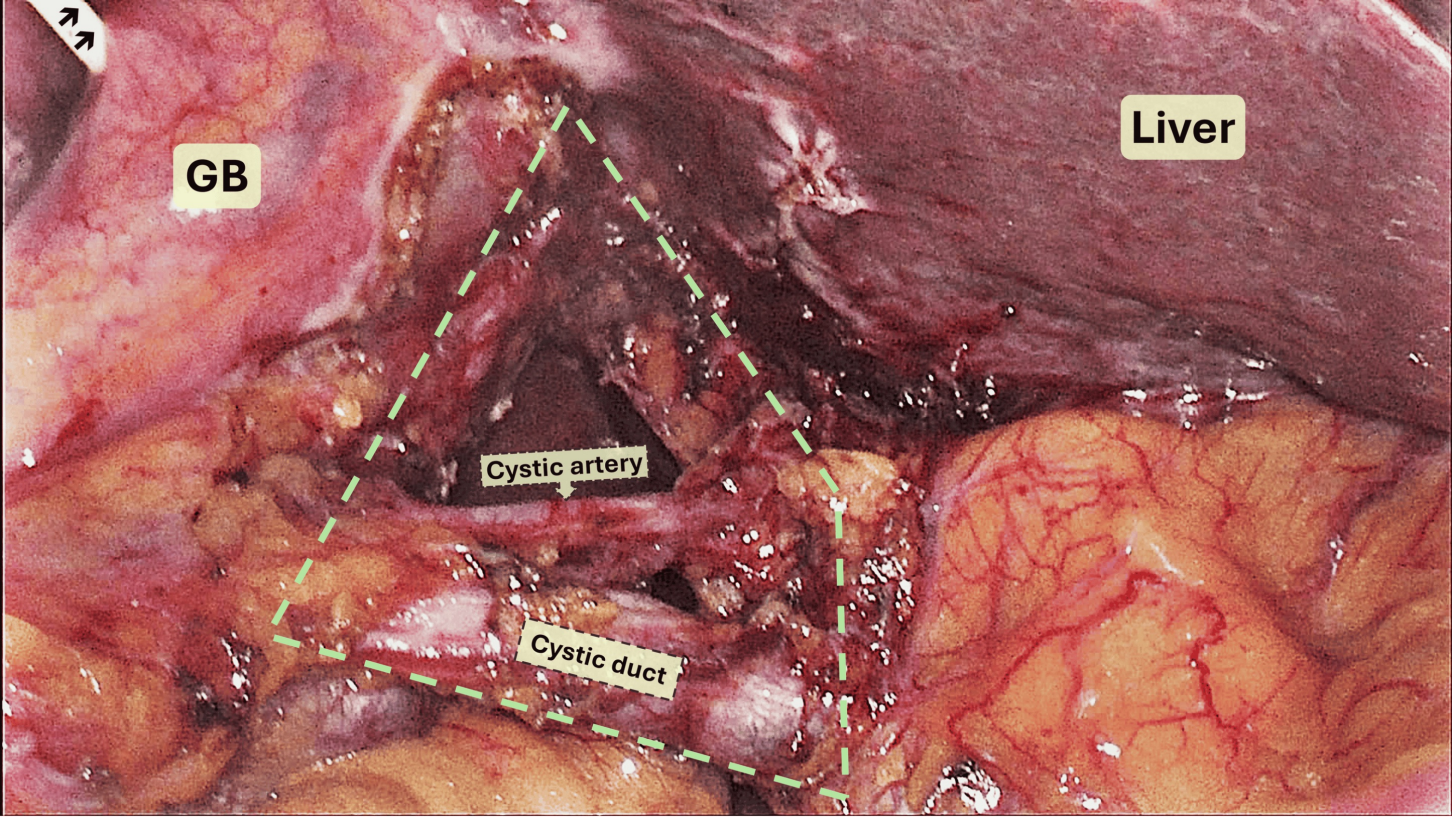
Anatomy of hepatocystic triangle Intraoperative image shows the hepatocystic triangle (yellow outline) after achieving the CVS: IOC catheter (arrow). In this case, the inner border of the hepatocystic triangle (common bile duct) is not visualized; instead, it may represent the aberrant RPHD as seen on IOC. CVS: critical view of safety; IOC: intraoperative cholangiography; RPHD: right posterior hepatic duct; GB: gallbladder

**Figure 3:**
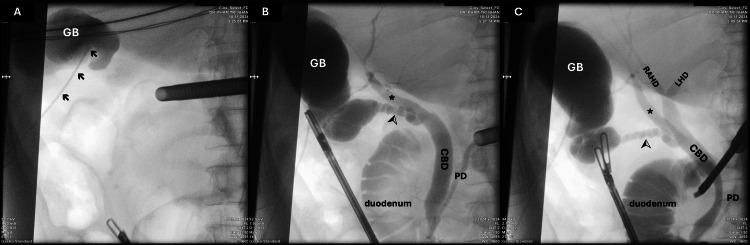
Intraoperative cholangiography via gallbladder A: Contrast agent is filled into the gallbladder (GB) by direct injection into the body through a catheter (arrow). B: Contrast agent shows the cystic duct (arrowhead) and the variant of the aberrant RPHD (asterisk) that drains directly into the common bile duct (CBD). C: The right anterior and left hepatic ducts (RAHD-LHD) are visible after blocking the end of the CBD. RPHD: right posterior hepatic duct; RAHD: right anterior hepatic duct; LHD: left hepatic duct; PD: pancreatic duct

The patient was managed conservatively for acute pancreatitis with bowel rest, intravenous fluids, and analgesia. Following clinical stabilization, elective laparoscopic cholecystectomy was planned to address the underlying cholelithiasis and prevent recurrent pancreatitis. During surgery, a standard four-port laparoscopic technique was employed. Upon dissection of Calot's triangle, the anatomical variation noted on MRCP was confirmed. The CVS technique was utilized to ensure safe identification of biliary structures (Figure 2). IOC through the gallbladder was performed to further delineate the biliary anatomy, which corroborated the aberrant drainage of the cystic duct into the RPHD (Figure 3). The cystic duct was carefully ligated and divided, and the gallbladder was removed without complications.

Postoperative course

The patient’s recovery was uneventful. He resumed oral intake on the first postoperative day and was discharged on the third postoperative day in stable condition. At the two-week follow-up, the patient reported complete resolution of symptoms. Laboratory tests conducted during the follow-up visit revealed normal liver enzyme levels (AST and ALT), indicating no ongoing hepatobiliary pathology. Histopathological examination of the gallbladder confirmed chronic cholecystitis with cholelithiasis.

## Discussion

Anomalies in the biliary anatomy are not uncommon, with studies suggesting that anatomical variations in the biliary tree occur in approximately 28-47% of individuals​ [6,7]. Among these, variations involving the RPHD draining into the common bile duct (CBD) and the cystic duct entering the RPHD are exceedingly rare, with a reported prevalence ranging from 0.9% to 4%​ [6,8]. This configuration represents a high-risk variant due to its proximity to critical structures and its potential to disrupt normal anatomical landmarks.

The significance of such variations becomes evident during laparoscopic cholecystectomy, where anatomical misidentification is the primary cause of iatrogenic bile duct injuries. Way et al. (2003) reported that 97% of these injuries result from a visual misperception, often exacerbated by atypical biliary anatomy, inflammation, or scarring at the hepatocystic triangle​ [9]. Misidentification in cases of RPHD-CBD variation poses a dual threat; it not only risks inadvertent ligation or transection of the aberrant duct but also complicates intraoperative decision-making when faced with unexpected anatomy​ [10,11]. Inflammation and adhesions further compound the challenge by obscuring visual landmarks and distorting the biliary anatomy. These conditions are common in patients presenting with acute or chronic cholecystitis, which often coexists with such anomalies. In a meta-analysis of biliary injuries, aberrant anatomical patterns were significantly associated with higher rates of complications, underscoring the importance of recognizing these variations preoperatively​ [10,11]. Thus, identifying and addressing these variations preoperatively through imaging modalities like MRCP or intraoperatively with strategies such as the CVS or IOC is essential to minimizing risks and ensuring patient safety.

MRCP plays a pivotal role in the preoperative assessment of biliary anatomy, particularly in identifying rare cystic duct variants and aberrant hepatic ducts [12]. These anatomical variations, though uncommon, are clinically significant due to their association with an increased risk of BDI during laparoscopic cholecystectomy [13,14]. In our case, initial CT revealed an unusual configuration at the biliary confluence, raising suspicion of a ductal anomaly. This prompted further evaluation with MRCP, which successfully demonstrated a distinct anatomical variation; the cystic duct joined directly with an aberrant RPHD, forming a common channel before draining into the common bile duct, without confluence with the right anterior hepatic duct (RAHD) or a unified right hepatic duct (RHD). This preoperative identification of the aberrant anatomy enabled tailored surgical planning, including a deliberate dissection strategy and the use of IOC to confirm the anatomy before transection. MRCP thus provided a non-invasive, high-resolution biliary roadmap that directly contributed to the safety of the procedure [4]. Given its diagnostic accuracy and ability to delineate complex biliary variants, MRCP should be strongly considered when preoperative imaging suggests abnormal ductal confluence.

The CVS, first described by Strasberg, plays a pivotal role in ensuring biliary safety during laparoscopic cholecystectomy. It consists of three essential steps: clearing the hepatocystic triangle of fat and fibrous tissue, dissecting the lower third of the gallbladder off the liver to expose the cystic plate, and identifying only two tubular structures entering the gallbladder before division​ [15]. In our case, despite the presence of a rare biliary anomaly where the cystic duct inserted into the RPHD, the application of CVS alone allowed for a safe dissection. However, as demonstrated in similar cases classified as Huang type A5 - where the RPHD aberrantly drains into or receives drainage from the cystic duct - the CVS may be insufficient to prevent BDI if aberrant ducts are misidentified as the cystic duct [1,2,16]. The anatomical complexity in such variants poses a significant risk of inadvertent transection of a major sectoral duct, potentially leading to severe complications such as bile leakage or hepatic atrophy. Reports have documented instances where CVS was achieved technically, yet unrecognized biliary anomalies led to major injuries​ [10]. Therefore, in cases with suspected or confirmed ductal variations, particularly involving the right posterior sectoral duct, CVS must be interpreted with caution. Adjuncts such as IOC or ICG fluorescence cholangiography may enhance biliary mapping and reduce the risk of injury​ [17]. In our case, IOC was employed via the gallbladder and proved effective in delineating the aberrant anatomy, reinforcing the importance of multimodal strategies even when CVS is achieved.

IOC plays a pivotal role in visualizing biliary anatomy during cholecystectomy, particularly in cases involving anatomical variations [17]. Routine use of IOC has proven to enhance the safety and accuracy of biliary surgery by providing real-time visualization of the biliary tree, with reported success rates of up to 93.3% [18]. In this case, IOC performed via the gallbladder, rather than the cystic duct, emerged as a safer and more effective technique. This approach bypassed the technical challenges and risks of cystic duct cannulation, such as damage to adjacent structures like the aberrant RPHD, which is at particular risk in configurations where the RPHD and cystic duct form a common trunk before joining the common bile duct. By directly injecting contrast through the gallbladder, this method minimized the risk of injury while simplifying the procedure, especially in the presence of inflammation or distorted anatomy [19]. Furthermore, the success rate of this approach has been shown to be comparable to, if not higher than, traditional cystic duct cannulation [19]. However, it is important to note that IOC via the gallbladder may not delineate finer details of distal biliary structures as clearly, and its interpretation requires surgical expertise [20]. Despite these limitations, the use of gallbladder IOC in this case effectively mitigated risks associated with aberrant biliary anatomy and provided reliable imaging, highlighting its potential as a preferred approach in challenging anatomical scenarios.

This case report has several limitations. First, ultrasound images were not available for inclusion due to data retrieval constraints, which may limit the visual documentation of the initial diagnostic process. Second, as with all single-patient case reports, the findings described herein may not be generalizable to broader clinical practice. Finally, long-term follow-up data beyond the early postoperative period were not available, which limits our ability to assess potential delayed complications or outcomes associated with this anatomical variation.

## Conclusions

This case report underscores the pivotal role of preoperative imaging and intraoperative strategies in managing rare anatomical variations of the biliary tree. The identification of the cystic duct draining into an aberrant RPHD was facilitated by MRCP, highlighting its utility in preoperative planning. During surgery, employing the CVS and IOC ensured precise anatomical delineation and prevented iatrogenic BDI. The identified anatomical variant was safely managed, and the postoperative course was uneventful, reinforcing the practical success of this approach.

The findings reinforce the importance of integrating advanced imaging modalities and standardized surgical protocols, such as CVS, particularly in the context of complex biliary anatomy. Furthermore, the safe and effective use of IOC demonstrates its value in enhancing intraoperative decision-making, even in resource-limited settings. Future studies could explore the potential of emerging technologies, such as ICG fluorescence cholangiography, to further optimize the management of challenging biliary variations. By adhering to these evidence-based strategies, surgeons can achieve optimal outcomes while minimizing the risks of bile duct injury and postoperative complications.
